# R-spondin2, a novel target of NOBOX: identification of variants in a cohort of women with primary ovarian insufficiency

**DOI:** 10.1186/s13048-017-0345-0

**Published:** 2017-07-25

**Authors:** Justine Bouilly, Isabelle Beau, Sara Barraud, Valérie Bernard, Brigitte Delemer, Jacques Young, Nadine Binart

**Affiliations:** 1 Inserm U1185, Faculté de Médecine Paris Sud 63, Univ Paris Sud, Université Paris-Saclay, rue Gabriel Péri, 94276 Le Kremlin-Bicêtre Cedex, France; 2 Service d’Endocrinologie-Diabète-Nutrition, CHU de Reims-Hôpital Robert-Debré, 51092 Reims, France; 3 APHP, Hôpital de Bicêtre, Service d’Endocrinologie et des Maladies de la Reproduction, F-94275 Le Kremlin-Bicêtre, France

**Keywords:** Primary ovarian insufficiency (POI), *RSPO2*, Folliculogenesis, Genetic variants

## Abstract

**Background:**

R-spondin2 (Rspo2) is a secreted agonist of the canonical Wnt/β-catenin signaling pathway. Rspo2 plays a key role in development of limbs, lungs and hair follicles, and more recently during ovarian follicle development. *Rspo2* heterozygous deficient female mice become infertile around 4 months of age mimicking primary ovarian insufficiency (POI). The study aimed to investigate the regulation of *RSPO2* and its potential involvement in pathophysiology of POI.

**Methods:**

We cloned the *RSPO2* promoter and performed transcriptional assays to determine if *RSPO2* can be regulated by NOBOX, an ovarian transcription factor. Then, we evaluated 100 infertile women after obtaining a detailed history of the disease and follicle-stimulating hormone measurements, besides karyotype determination and fragile-X premutation syndrome investigation. All exons, intron-exon boundaries and untranslated regions of the *RSPO2* gene were identified by sequencing, and the results were statistically analyzed.

**Results:**

We found that RSPO2 can be regulated by NOBOX via the presence of NOBOX Binding Element in its promoter. Among 9 identified variants in POI women, 4 of them were equally homozygous, 4 have never been described (c.-359C > G, c.-190G > A, c.-170 + 13C > T and c.-169-8 T > A), only one c.557 T > C was predicted to alter a single amino acid in the RSPO2 protein (p.Leu186Pro).

**Conclusions:**

*RSPO2* is a novel target gene of the NOBOX key transcription factor, confirming its important role during the follicular growth in ovary. However, *RSPO2* mutations are rare or uncommon in women with POI.

## Background

Primary Ovarian Insufficiency (POI) is an important human disease model that provided substantial understanding into the factors involved in differentiation and ovarian development. This rare disease is characterized by amenorrhea with elevated gonadotropin levels, and affects 1% of women before the age of 40 years [1]. POI encompasses a heterogeneous spectrum of conditions, through two major mechanisms, follicle dysfunction and follicle depletion [2]. Although, the majority of cases remain idiopathic, POI can be triggered by autoimmune disease, viral, iatrogenic or genetic causes [1]. The disorder may be associated in syndromic diseases, such as Turner’s syndrome, X-Fragile syndrome or BPES syndrome [3]. Genetic component such as X-chromosome abnormalities, deletions, *FMR1* premutations, *BMP15* variants, were identified as the first genetic causes of the pathophysiology [4]. To date, about 30 autosomal genes were identified as involved in the pathogenesis of POI, and more recently a digenic form has been observed in large cohort of POI women [5]. For instance, recent publications reported mutations in genes involved in meiosis like *STAG3* [6], *HFM1* [7] or *SYCE1* [8] and in transcription factors including *FIGLA* [9] or *SOHLH2* [10]. However, except the high prevalence of *NOBOX* [11] and *BMP15* mutations [4] no gene has been shown implicated in more than 6% and 3%, respectively, of POI cases.

Most POI are idiopathic, thus it seems to be important to research additional factors. Potential candidates could be members of R-spondin (RSPO) family that are ligands activating Wnt pathway [12]. Among them, R-spondin1 acts on ovarian differentiation and *RSPO1* mutations have been found in patients with sex differentiation disorders [13]. It has recently been suggested that Rspo2, another member of this family, could be important in regulating ovarian follicle development [14]. Rspo2 is expressed in mouse oocytes from primary follicles to growing follicles. An ex vivo and in vivo treatment of mouse ovary with Rspo2 promoted development of follicles. Moreover, *Rspo2* heterozygous deficient female mice become infertile around 4 months of age, then mimicking POI [15–17].

So, the aim of this study was to analyze *RSPO2* regulation by NOBOX, a master-transcription factor of ovarian folliculogenesis and to determine whether *RSPO2* genetic variation is associated with idiopathic POI women.

## Methods

### Cloning of *RSPO2* promoter and luciferase assays

*RSPO2* promoter containing two Nobox Binding Elements (NBE, region −3560 to −2606) was obtained by PCR and subcloned into SacI and XhoI sites of pGL4.26 Luc/miniP vector (Promega). COS7 cells seeded on 96-well plates were transfected with plasmids encoding the human wild-type (WT) or R303X-NOBOX transcription factor, firefly luciferase under control of the *RSPO2* promoter, and Renilla luciferase (internal control) as previously reported [11] This R303X-NOBOX mutant is devoid of a large part of the homeodomain and used as a negative control [11]. Firefly and Renilla luciferase activities were measured consecutively with dual-luciferase assays (Promega) and a TriStar reader (Berthold), and are expressed as relative light units. Each assay was performed independently three times and included six replicates.

### Statistical analysis

Statistical analysis was performed using PRISM 5 software (GraphPad). Data were analyzed using one-way ANOVA with Kruskal-Wallis post-tests. Data are shown as mean ± SEM. (**), *p* value < 0.01 and (****), *p* < 0.0001.

### Patients

One hundred idiopathic POI women were recruited following Institutional Review Board approval (reference PHRC No. A0R03 052, approved by Bicêtre Ethical committee (CPP Ile-de-France VII #PP 16–024). All the participants gave their written informed consent for hormonal and genetic analyses, in keeping with the provisions of the French Bioethics Law and the Declaration of Helsinki and after approval by the Bicêtre and Reims Hospital ethic committees. Inclusion criteria were cessation of menstrual cycles for more than 4 months before 40 years of age, with at least two serum FSH concentrations of >30 IU/L. No Turner syndrome, X chromosome caryotypic anomalies, neither *FRM1* premutation were present in the patients. Autoimmune and metabolic causes (such as galactosemia) of POI were excluded in this series. As a control population, 100 healthy women, 20–39 years of age were included in the present study belonging to the VARIETE cohort which was an open, prospective, national, multicenter, nonrandomized study of healthy volunteers, designed to establish normative data for hormones (ClinicalTrials.gov Identifier: NCT01831648). All women had normal age of puberty onset, body mass index, and regular spontaneous menstrual cycles (27–31 days). None had clinical signs that could suggest POI. All healthy subjects gave their written informed consent to participate in the study, which was approved by the Paris-Sud Ethics committee before the beginning of the study.

### Hormonal measurements

FSH and LH plasma levels were measured by radioimmunoassay (Immunotech Beckman Coulter, France) as previously reported [18]. Estradiol was determined after previous plasma extraction (DiaSorin, Italy; Schering CisBio International, France; and Immunotech Beckman Coulter, respectively) [19]. Serum inhibin B levels were measured with a commercial ELISA (Serotec, Kidlington, Oxford, United Kingdom) with a detection limit of 6 ng/L and inter- and intraassay coefficients of variation of 13 and 6%, respectively (at 15 ng/L). Serum AMH was measured by an enzymatically amplified two-site immunoassay (AMH Gen II ELISA; Beckman Coulter, Marseilles, France). All steps of procedure were conducted according to the manufacturer’s recommendations. The lower detection limit of the assay was 0.7 pmol/L. The mean interassay coefficient of variation (CV) was 8.6%, and the mean intraassay CV was 4.0% at 22 pmol/L.

### DNA sequencing and mutational analysis of genomic DNA

Genomic DNA was isolated from human blood leukocytes using standard methods, and the exons of the *RSPO2* gene were sequenced in both directions in 100 women with POI. Reference sequences were obtained from Ensembl (ENSG00000147655 for genomic DNA, ENST00000276659 for messenger RNA) and the National Center for Biotechnology Information for messenger RNA NM_178565.4.

Mutational analysis of genomic DNA was studied by sequencing all six exons and exon–intron boundaries after amplification of *RSPO2* gene. The entire coding sequence and intron-exon boundaries were amplified from genomic DNA using 6 sets of primers (Table 1). After a 5-min hot start, DNA was amplified for 35 cycles (95 °C for 45 s, 56–65 °C for 1 min, and 72 °C for 45 s) in 1× buffer with 2.5 mM MgCl_2_, 0.2 mM dNTPs, 0.06 μM concentrations of each primer, 100 ng of genomic DNA, and 1.25 U of Dream Taq (Fermentas) in a total volume of 50 μL. The amplified samples were subjected to agarose gel electrophoresis to ensure single bands. PCR products were purified, then sequenced on an automated sequencer (3100 Applied Biosystems) using the same primers as for PCR by Sanger Method.

**Table 1:** Primers used for PCR

Location	Primer sequence
Exon 1	F: CCTAGACTTAGATGCCTTG
	R: GGTGTGGGTTGCCTAC
Exon 2	F: GAGGTTGCTAATTCACTGAT
	R: AGGGTACAGAAAACAGAGTG
Exon 3	F: TGAGTTTCCTCTTTGTTTCT
	R: TTCAAAATCTTCAACTTAGC
Exon 4	F: AAAGAGACAGGGATGACTTA
	R: TAGCAAATTTTACAGCAAGA
Exon 5	F: CCAAAAGGTGAGTATAGGTC
	R: GCACTTCATATTTTTCACAA
Exon 6	F: CAGACAGAGCTAACCAATAA
	R: TGGTAGTAGCTTCTTCAGTG

F: Forward; R: Reverse

Nucleotide numbering reflects cDNA numbering (http://www.HGVS.org/varnomen) with +1 corresponding to the A of the ATG translation initiation codon (codon 1) in the reference sequence.

Ethnically-matched controls from the 1000 Genomes Project Database [20] and the Exome Aggregation Consortium [21] were used for individual variant and gene mutation frequencies in this study.

### Molecular modeling

PolyPhen (Polymorphism Phenotyping, http://genetics.bwh.harvard.edu/pph2/; http://www.HGVS.org/varnomen) and Sorting Intolerant From Tolerant (SIFT; http://sift.bii.a-star.edu.sg/) software packages as well as evolutionary conservation were used to predict the pathogenic nature of the coding sequence alteration.

Alternative Splice Site Predictor (ASSP) (http://wangcomputing.com/assp/), Netgen2 (http://www.cbs.dtu.dk/services/NetGene2/), NNSplice.v.0.9 (http://www.fruitfly.org/seq_tools/splice.html), Human Splicing Finder v.2.4.1 (http://www.umd.be/HSF/) software were used to predict splicing site of intronic variations.

## Results

### *RSPO2 is a* novel target gene of NOBOX

**Fig. 1:**
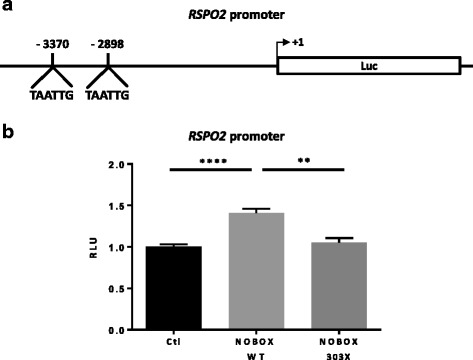
Identification of RSPO2 as a NOBOX target and in vitro assay of NOBOX transcriptional activity using *RSPO2* promoter as reporter gene. **a** Schematic map of 954 bp promoter sequence of human *RSPO2* including 2 NOBOX binding elements (NBE) located at −3370 and −2898 upstream of the transcription site. **b** NOBOX activity is shown as the luciferase activity above baseline, which is defined as the activity observed in cells transfected with empty vector (Ctl). The transcriptional activity of wild-type NOBOX (NOBOX WT) was studied using *RSPO2* promoter transfected in COS7 cells. As a negative control, the NOBOX deleted homeodomain mutant (NOBOX-303X) was used. Results are presented as mean ± SEM of 3 independent experiments each performed in sixplicate. **P < .01 ****P < .0001. RLU, relative light units

The ovarian *Rspo2* expression was reported very low in *Nobox*-null mice suggesting that it is controlled by this transcription factor [22]. In silico analysis of *Rspo2* promoter revealed the presence of two NOBOX Binding Element (NBE) (Fig. 1a). A 954 bp fragment of the human *RSPO2* promoter, containing two NBE, was cloned in a luciferase reporter plasmid (*RSPO2*-promoter).

To measure the effects of NOBOX on *RSPO2* transactivation promoter, COS7 cells were transfected with *RSPO2*-promoter and wild-type (WT)-NOBOX or R303X-NOBOX, a mutant devoid of a large part of the homeodomain find in POI patient [11]. We observed a significant increased activation of *RSPO2*-promoter with WT-NOBOX suggesting that NOBOX can bind *RSPO2* promoter (Fig. 1b). As a negative control R303X-NOBOX was unable to activate the promoter (Fig. 1b).

### Clinical characteristics of the patients

One hundred POI patients completed the study. Median age was 30 (13–39) at the time of the diagnosis. Clinical and hormonal characteristics of these patients are summarized in Table 2. Most of the patients (*n* = 80) presented with normal puberty and secondary amenorrhea whereas 20 presented with primary amenorrhea with variable pubertal development (from absence (5), partial (11) to complete development (4)). Among patients with secondary amenorrhea, symptoms appear from 15 to 39 year-old with a median age at 29.

**Table 2:** Clinical and hormonal characteristics of 100 POI patients

Population studied			Hormonal evaluation at diagnosis				
Amenorrhea	n	Median age at diagnosis (range)	FSH [SD] (range) (IU/L)	LH [SD] (range) (IU/L)	Estradiol [SD] (range) (pmol/L)[	Inhibin B [SD] (range) (ng/L)	AMH [SD] (range) (pmol/L)
Primary	20	18 years (13–32)	70.0 [33] (20–164)	35.1 [18.7] (9–100)	52.8 [60.5] (0–234)	14 [9.5] (0–42)	1.3 [2.0] (0–11)
Secondary	80	29 years (15–39)					
Normal range of basal levels in controls			(3–9)	(1–5)	(73–1284)	(60–200)	(15.7–48.5)

### Identification of SNPs in the *RSPO2* locus

**Fig. 2:**
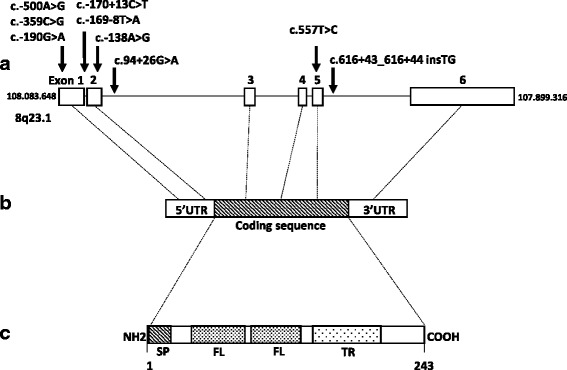
Schematic representation of *RSPO2* gene and RSPO2 protein. **a** *R-spondin2* gene contains 6 exons (reference sequence was based on NC_000008.11 and ENSG00000147655). Locations of 9 variations are indicated by an arrow. **b** *R-spondin2* mRNA includes exons 2 to 6 encoding the protein (reference sequence was based on NM_178565 and ENST00000276659). **c** RSPO2 encompasses a signal peptide (SP), two cysteine-rich furin like repeats (FL) and a thrombospondin type 1 repeat domain (TR) (reference sequence was based on NP_848660)

Analysis of the *RSPO2* gene in 100 POI patients revealed the presence of 9 sequence variations identified in the non-coding and coding portions (Fig. 2 and Table 3). All 9 variations were heterozygous and 4 of them were equally homozygous. Among them, 4 of these SNPs have never been described (c.-359C > G, c.-190G > A, c.-170 + 13C > T and c.-169-8 T > A). Of the 9 identified SNPs, only one c.557 T > C was predicted to alter a single amino acid in the RSPO2 protein (p.Leu186Pro). This variant was found heterozygous in 51% and homozygous in 20% of patients and was also described as very common in controls by Exome variant server and the 1000 Genomes. It is predicted as non-deleterious by Polyphen and SIFT softwares and hence clearly could be considered as not disease-causing.

**Table 3:** *RSPO2* variations in 100 women with POI

Sequence variation	Location	Amino Acid variation	dSNP identifier	Exome Genome Variant (%)	1000 Genomes (%)	Allele frequency in patients (%)	
						heterozygote	homozygote
c.-500 A > G	Exon 1	0 (5’UTR)	rs55916111	/	4.4	10	4
c.-359 C > G	Exon 1	0 (5’UTR)	/	/	/	2	0
c.-190 G > A	Exon 1	0 (5’UTR)	/	/	/	1	0
c.-170 + 13C > T	Intron 1	0 (intron)	/	/	/	1	0
c.-169-8 T > A	Intron 1	0 (intron)	/	/	/	2	0
c.-138 A > G	Exon 2	0 (5’UTR)	rs112769314	/	2.1	1	0
c.94 + 26 G > A	Intron 2	0 (intron)	rs10955475	18.7	21.4	23	2
c.557 T > C	Exon 5	L186P	rs601558	89.7	46.3	51	20
c.616 + 43_616 + 44 insTG	Intron 5	0 (intron)	rs35069883	89.4	46.1	51	20

Note: dSNP are Single Nucleotide Plymorphism

Nucleotide numbering reflects cDNA numbering with +1 corresponding to the A of the ATG translation codon in the reference sequence (NC_000008.11)

Otherwise, 4 variations (c.-170 + 13C > T, c.-169-8 T > A, c.94 + 26G > A and c.616 + 43_616 + 44insTG) were found in intron-exon junction. The in silico analysis using Alternative Splice Site Predictor (ASSP), Netgen2, NNSplice.v.0.9, Human Splicing Finder v.2.4.1 did not display the generation of a new splicing site. The remaining 4 variations located in 5’UTR non coding sequence (c.-500A > G, c.-359C > G, c.-190G > A and c.-138 A > G) were rare and did not cause the production of a new start codon (ATG).

## Discussion

More than 80% of POI causes are unknown until now, it is of interest that multiple approaches are required to discover new responsible candidate genes. Mouse forward genetics is a powerful tool for the identification of genes and pathways important in biological process and has been very valuable for the characterization of key players in ovarian folliculogenesis [23]. Recently transgenic mouse studies supported the role of *Rspo2* in follicle development. Human *RSPO2* gene is located on chromosome 8 and encodes a member of the R-Spondin family of secreted proteins involved in β-catenin activation through the canonical Wnt pathway leading to T-cell factor-dependent gene activation [24–26]. *Footless* mutant mice with a transgene insertion in *the Rspo2 gene* showed many malformations [15], although *Footless* heterozygous animals were indistinguishable from wild-type animals through weaning, female animals were only fertile until 4 months of age. Similarly, heterozygous *Rspo2* mutant female mice were 25% sterile at 4 months and 85% sterile at 5 months [25]. This infertile phenotype associated with the oocyte *Rspo2* expression and follicular growth function led us to consider this gene as a possible candidate gene in POI. It is likely that the initial waves of follicle growth were maintained in *Rspo2* heterozygous mutants, but inappropriate levels of Rspo2 in oocytes eventually led to the failure of follicle development during late reproductive life. It was recently shown that the administration of Rspo2 agonist promotes human early follicle development after xenografted ovarian cortical pieces in immunodeficient mice [14]. Loss of function mutations in *RSPO2* has not previously been associated with any phenotype in women.

Of interest, we described a number of mutations of the oocyte-specific homeobox gene *NOBOX* associated with POI patients [11, 27]. Interestingly, the ovarian *Rspo2* expression was reported very low in *Nobox*-null mice [22]. Otherwise, by an in silico analysis of *RSPO2* promoter the presence of NOBOX Binding Element (NBE) is revealed. Here, the transcriptional assays showed that NOBOX was able to activate the *RSPO2* expression. This suggests that *RSPO2* is a novel target gene of NOBOX, a master regulator of the folliculogenesis. It was, thus, of interest to look at *RSPO2* variants and to evaluate the role of RSPO2 in POI patients.

Among 100 independent idiopathic POI patients, we identified 9 Single Nucleotide Polymorphisms (SNPs) in *RSPO2*, four of them had never been described and one caused an amino-acid change but none of them seems to be deleterious when analyzed in silico*.*

The variations of *RSPO2* gene found in this cohort seem to be polymorphisms with current technics, and *RSPO2* mutations are therefore rare. However, RSPO2 appears to have an important role in ovary and it should be interesting to search *RSPO2* mutations in a larger cohort of POI patients. Moreover, RSPO2 is also a local intraovarian factor expressed at specific stages of follicular development. Taking into account, it would be rare to find a pathogenic variant or polymorphism in the peripheral blood of patients with POI. The disruption mediated through RSPO2 is most likely a local disruption which is temporally associated with folliculogenesis. Since it is challenging to study RSPO2 variations in ovarian tissue, we cannot exclude some somatic variants. Identically, the search of mutations in genes largely involved in different folliculogenesis steps such as *NANOS3, FOXO3a and PRLR* [28–30] has also been performed without any success. Moreover, many factors and regulators involved in folliculogenesis are intraovarian and work in a paracrine manner. The expression in oocytes and granulosa cells is more important than in somatic cells. Therefore, the paper demonstrating that R-spondin2 acts in folliculogenesis [14] and the present study support and reinforce the importance of RSPO2 in this process.

Future high-throughput sequencing of such genes in women with idiopathic ovarian insufficiency should provide a better idea of the contribution that oocyte genes make to nonsyndromic POI.

## Conclusion

RSPO2 is described as a key actor of the folliculogenesis regulation. In the present study, we demonstrated that *RSPO2* is regulated by NOBOX a master transcription factor, suggesting that *RSPO2* can be mutated in women affected by primary ovarian insufficiency. However, our analysis has not allowed to detect *RSPO2* pathogenic variants. We conclude that mutations in the coding regions of *RSPO2* are not common causes in POI.

## Abbreviations

AMH: Anti-Müllerian hormone
DNA: deoxyribonucleic acid
FSH: Follicle Stimulating Hormone
LH: Luteinizing Hormone
NBE: Nobox Binding Element
POI: primary ovarian insufficiency
Rspo: R-spondin
SNP: Single nucleotide polymorphism
WT: wild-type
